# Phenolic-Rich Extracts from Circular Economy: Chemical Profile and Activity against Filamentous Fungi and Dermatophytes

**DOI:** 10.3390/molecules28114374

**Published:** 2023-05-26

**Authors:** Andrea Lombardi, Margherita Campo, Pamela Vignolini, Marco Papalini, Mirco Pizzetti, Roberta Bernini

**Affiliations:** 1 Department of Agriculture and Forest Sciences (DAFNE), University of Tuscia, Via San Camillo de Lellis, 01100 Viterbo, Italy; andrea.lombardi@unitus.it; 2 Phytolab, Department of Statistics, Informatics, Applications “G. Parenti”, DiSIA, University of Florence, Via Ugo Schiff 6, 50019 Sesto Fiorentino, Italy; margherita.campo@unifi.it (M.C.); pamela.vignolini@unifi.it (P.V.); 3 Bioricerche S.r.l., Loc. Ferro di Cavallo, 58034 Castell’Azzara, Italy; direzione@bioricerche.com (M.P.); mirco.pizzetti@bioricerche.com (M.P.)

**Keywords:** phenolic-rich extracts, circular economy, green chemistry, antifungal activity, filamentous fungi, dermatophytes

## Abstract

Fungal infections represent a relevant issue in agri-food and biomedical fields because they could compromise quality of food and humans’ health. Natural extracts represent a safe alternative to synthetic fungicides and in the green chemistry and circular economy scenario, agro-industrial wastes and by-products offer an eco-friendly source of bioactive natural compounds. In this paper, phenolic-rich extracts from *Olea europaea* L. de-oiled pomace, *Castanea sativa* Mill. wood, *Punica granatum* L. peel, and *Vitis vinifera* L. pomace and seeds were characterized by HPLC-MS-DAD analysis. Finally, these extracts were tested as antimicrobial agents against pathogenic filamentous fungi and dermatophytes such as *Aspergillus brasiliensis*, *Alternaria* sp., *Rhizopus stolonifer*, and *Trichophyton interdigitale*. The experimental results evidenced that all extracts exhibited a significant growth inhibition for *Trichophyton interdigitale*. *Punica granatum* L., *Castanea sativa* Mill., and *Vitis vinifera* L. extracts showed a high activity against *Alternaria* sp. and *Rhizopus stolonifer*. These data are promising for the potential applications of some of these extracts as antifungal agents in the food and biomedical fields.

## 1. Introduction

Fungal infections pose a major challenge to academia and industry operating in agri-food and biomedical fields. Annually, fungal pathogens can lead to losses of up to 30% in crops and orchards, endangering the quality and safety of food and feed even through the production of mycotoxins such as aflatoxins by *Aspergillus*, which represent a serious threat for humans due to their carcinogenicity and large diffusion among different food matrices [1,2,3].

Fungi are major concerns throughout the entire food supply chain, including post-harvest phases, where losses can lead to resources depletion, increased safety risks for consumers, and waste production [4]. The occurrence across supply chains of microbial pathogens, including fungi and the consequent insurgence of food-borne diseases, could be mitigated and prevented through the application of food safety principles according to the Hazard Analysis and Critical Control Point (HACCP) methodology and ISO 22000:2018 standard [5,6,7]. The presence of yeasts and fungi may be investigated in food and feed by certified laboratories according to standard horizontal ISO methods [8,9].

Aside from food contamination, humans can be affected by fungi even by inhalation, leading to the onset of life-threatening illness, especially in immunocompromised subjects, such as aspergillosis, allergies, and asthma [10,11]. In addition to invasive diseases, cutaneous infections could be caused by fungal species and dermatophytes responsible for a series of human disorders of nails, hair, and skin due to their ability to degrade keratin [12]. The increased tolerance and resistance to synthetic antifungal agents are reflected in the enhancement of morbidity rates [13].

Within this framework, natural compounds could offer concrete opportunities for the development of novel antifungal agents to fight fungal pathogens affecting humans, animals, and foods in compliance with the principles of sustainability and green chemistry [14,15,16,17]. Among natural compounds, polyphenols represent an interesting group of secondary metabolites widely diffused in the plant world. They include several thousand compounds with a huge variability of molecular weight as phenolic acids, stilbenes, flavonoids, lignans, and tannins deriving from the acetate and shikimate pathways [18]. These compounds have multiple physiological activities: they act as defense agents against various abiotic and biotic stresses and in response to pathogen attacks, are signaling compounds, attract pollinating insects, are responsible for the color of flowers and some fruits, protect against UV-Vis radiation, and show structural functions [18]. They are also well known for their beneficial effects on human health in the prevention and treatment of chronic diseases such as diabetes, cancer, and cardiovascular and neurodegenerative diseases for their strong antioxidant, anti-inflammatory, antiaging, antiproliferative and antimicrobial activities [19,20,21,22,23].

Polyphenols are also found in food and agro-industrial by-products and wastes [24,25,26,27]. According to the green chemistry and circular economy concept, strategies aimed at the valorization of these materials should be designed and developed for the sustainability of production processes and the environment [28].

Olive (*Olea europaea* L.), chestnut (*Castanea sativa* Mill.), pomegranate (*Punica granatum* L.), and vine (*Vitis vinifera* L.) are typical cultivated plants in the Mediterranean area. Their stems, branches, leaves, and fruits contain polyphenols. Processing them produces large amounts of by-products and wastes that represent a cost to agri-business because they require proper disposal to ensure environmental sustainability. Alternatively, they can be valorized by recovering the active ingredients for use in various applications with significant economic and environmental benefits [28].

The production of extra-virgin olive oil from *Olea europaea* L. results in olive leaves, wastewaters, and pomace. These by-products and wastes contain phenolic alcohols and acids, and secoiridoids and flavonoids. Only 2% of the total polyphenols is found in olive oil; 53% and 45% are in wastewaters and pomace, respectively [29]. Chestnut (*Castanea sativa* Mill.) is mainly utilized for wood and fiber production. The corresponding wastes, rich in hydrolysable tannins with antioxidant and antimicrobial activity, are used as tanning agents for leather; mordants for textiles, paper, and wood; and as natural agents to clarify wine and stabilize the organoleptic characteristics [30,31]. Pomegranate juice production generates waste consisting mainly of peels and seeds. Peels constitute 50% of the total fruit and ellagitannins account for >99% of the total polyphenolic content of pomegranate. These compounds have shown promising therapeutic properties as anti-inflammatory and antibacterial agents [32,33]. Grape (*Vitis vinifera* L.) processing produces stalks, lees, marc, and grape seeds. The pomace accounts for about 20–30% of the original weight of the grapes, and the grape seeds for about 38–52% of the solid waste [34]. These by-products are valuable raw materials due to their high polyphenol content and antioxidant activity [35,36].

In this work, phenolic-rich extracts deriving from *Olea europaea* L. de-oiled pomace, *Castanea sativa* Miller wood, *Punica granatum* L. peel, and *Vitis vinifera* L. pomace and seeds were characterized by HPLC-DAD-MS to define the qualitative and quantitative profile of polyphenols. Finally, they were tested against a panel of pathogenic filamentous fungi and dermatophytes such as *Aspergillus brasiliensis* (ex *A. niger*), *Alternaria* sp., *Rhizopus stolonifer*, and *Trichophyton interdigitale*. The advantages of using these natural extracts over synthetic antifungal agents relate to the sustainability of the agri-food chain from a biorefinery perspective, according to green chemistry methodologies and the circular economy model.

## 2. Results and Discussion

### 2.1. Phenolic-Rich Extracts

Based on our experience on the valorization of agro-industrial by-products and wastes, *Olea europaea* L. de-oiled pomace, *Castanea sativa* Mill. wood, *Punica granatum* L. peel, and *Vitis vinifera* L. pomace and seeds were selected as starting materials for the corresponding phenolic-rich extracts named OEP, CSW, PGP, VVP, and VVS, respectively. OEP, CSW, and VVS were commercial industrial fractions obtained by sustainable processes through water or water–ethanol extraction and purification/concentration by membrane technology [35]. The low percentage of ethanol, in combination with water, increased the yield of extraction of polyphenols, avoiding high temperatures which would cause their degradation. This methodology allows for making the production processes sustainable even on an industrial scale, avoiding the use of pollutants or toxic solvents for both the extraction and refining steps. In particular, membrane technologies are currently an interesting example of a sustainable separation and concentration technology, often integrated in biorefineries to rationalize the processes and the structure of the plants, and to reduce environmental and economic impacts whilst maintaining the quality of the products. For the extracts under examination, several refining steps were applied from ultrafiltration up to reverse osmosis to obtain the concentrated solutions to be spray-dried and the purified water to be reused for subsequent extraction batches [37].

PGP and VVP were prepared in a laboratory as described in the Section 3 Materials and Methods.

OEP, CSW, PGP, VVP, and VVS were analyzed by HPLC-DAD-MS to define the qualitative and quantitative content of polyphenols (see Table 1 and Table 2 and Figures S1–S5; Tables S1–S5 in Supplementary Materials). As reported in Table 1, OEP, CSW, and PGP are characterized by a total polyphenol content of 173 ± 5 mg/g, 260 ± 3 mg/g, and 115 ± 2 mg/g, respectively. OEP contains mainly hydroxytyrosol (138 ± 4.0 mg/g) and tyrosol in lower amount (35.0 ± 0.8) [38]. The most representative phenolic compounds found in CSW and PGP are hydrolysable tannins such as vescalagin (47.6 ± 0.5) and castalagin (97.7 ± 0.9) in CSW, and α-punicalagin (27.1 ± 0.3) and β-punicalagin (58.5 ± 0.6) in PGP, together to low amounts of vescalin (9.3 ± 0.2), castalin (8.1 ± 0.2), α-punicalin (1.25 ± 0.04), and β-punicalin (1.32 ± 0.02) obtained by hydrolysis during water extraction [39,40,41,42].

As reported in Table 2, VVP and VVS are characterized by a total polyphenol content of 425 ± 8 mg/g and 686 ± 20 mg/g, respectively. They are represented by condensed tannins with different degrees of polymerization, which are stable under water extraction conditions. The main components are procyanidin tetramers with 293 ± 4 mg/g and 315 ± 9 mg/g in VVP and VVS, respectively. The monomers catechin and epicatechin were identified in both samples, although in different amounts (0.414 ± 0.008 mg/g and 0.320 ± 0.008 mg/g in VVP; 45 ± 1 mg/g and 30.3 ± 0.8 mg/g in VVS). The study of the chromatographic profile of VVP at 520 nm also allows for the identification and quantification of anthocyanins retained within the plant material after the winemaking process (total: 3.14 ± 0.07 mg/g). The absence of aglycones and large amounts of degradation products suggests that the transformation processes undergone by the plant material were able to keep most of the less stable compounds intact. However, the low amount of these compounds in a by-product such as grape marc is plausible, given that they are largely transferred into the wine during winemaking, and due to their low stability.

### 2.2. Antifungal Activity

As already reported in the Introduction, polyphenols showed antimicrobial activity and several phenolic-rich extracts have been tested against pathogens [43,44,45].

In this work, the selected fungi in relation to food and biomedical issues were *Alternaria* sp., *Aspergillus brasiliensis*, *Rhizopus stolonifer*, and *Trichophyton interdigitale*. *Alternaria* is a ubiquitous genus that includes about 300 species between saprophytes and pathogens [46]. Because of its wide diffusion in plants, it could represent a threat during pre- and post-harvest phases, even through the production of mycotoxins [47]. In particular, the *Alternaria* species can produce more than 70 different toxins, which led the European Food Safety Authority (EFSA) to reveal the exposure levels of the European population to *Alternaria* by-toxins, which were troubling owing to higher levels found on toddlers [48,49]. *Aspergillus brasiliensis* is one of the 18 species included in the black aspergilli group, *Aspergillus* section *Nigri* [50]. Generally confused with *A. niger*, this biseptate species is responsible for the production of several secondary metabolites such as deyhidrocarolic acid, funalenone, and malformins [51]. *Aspergillus brasiliensis* ATCC 16,404 is widely used as a reference microorganism in several European standard ENs for chemical disinfectants and antiseptics in the medical area [52]. Moreover, the food industry and international organizations use *Aspergillus brasiliensis* ATCC 16,404 as a test microorganism in bio validation assays, i.e., to investigate the effects of UV sterilization treatment on food packaging [53]. *Rhizopus stolonifer* is a Zygomycete which affects a wide range of fruits and vegetables, as well as bread. The etiological agent of “soft rot” and “black bread mold” is characterized by a fast penetration ability and the rapid growth of a hairy gray mycelium, resulting in one of the most uncontrollable postharvest pathogens [54]. *Trichophyton interdigitale* is a clonal anthropophilic line of *T. mentagrophytes* involved in human dermatophytosis such as athlete’s foot “tinea pedis” and onychomycosis [55,56]. As other *Trichophyton* sp. it may produce biofilms as a virulence factor, reducing the effectiveness of locally applied antifungal agents or making it necessary for them to be used at high concentrations [57].

In the experimental design of this work, for each pathogen three different concentrations of OEP, CSW, PGP, VVP, and VVS were tested (1.0%, 0.5% and 0.1% *w*/*v*) using a diffusion assay [58,59]. Even though, in the literature, higher concentrations are tested by diffusion assays (up to 5–10–15% *w*/*v*) [60,61,62], the maximum concentration evaluated in this work was 1%, both for technical reasons related to the influence of the extract on the technological properties of the PDA medium and for economic evaluation in view of the potential industrial application of extracts that should prove to be active. Benzoic acid (BA, E210) and potassium sorbate (SK, E202) were used as positive controls. These compounds are largely employed as food additives with antifungal properties. BA, in the limit of 0.1%, and SK are Generally Recognized as Safe (GRAS) by the Food and Drug Administration (FDA), and their use in Europe is regulated by EC 1333/2008 [63,64]. Moreover, BA is a component of Whitfield’s ointment, a topical treatment of tinea dermatophytosis still used today in developing countries [65,66].

**Figure 1:**
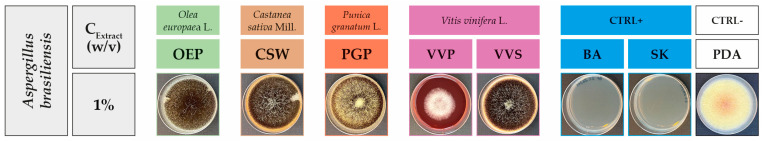
Antifungal activity of phenolic-rich extracts against *Aspergillus brasiliensis*.

**Figure 2:**
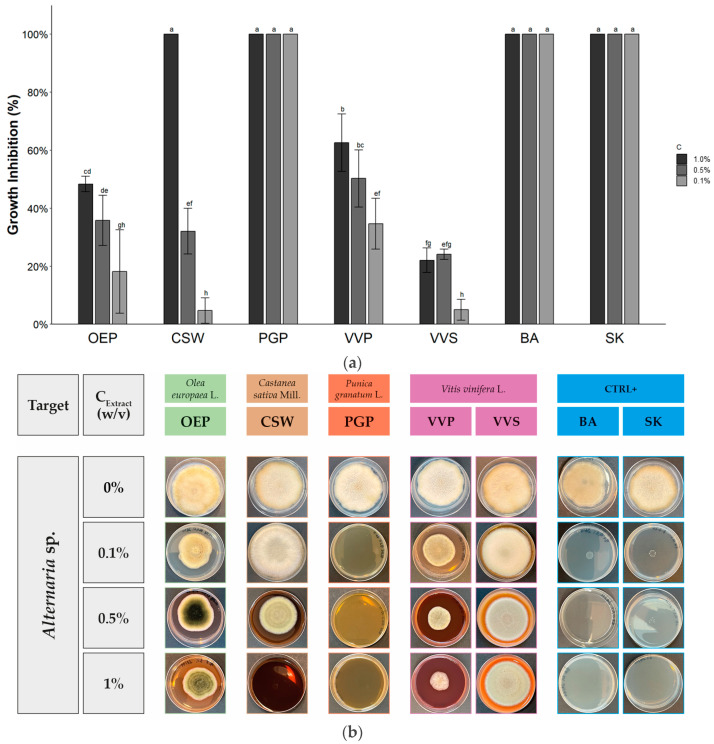
Antifungal activity of phenolic-rich extracts against *Alternaria* sp. (**a**) Histogram reporting the growth inhibitory activity of the extracts at different concentrations. Data expressed by mean ± SD (*n* = 6). Bars with different letters are significantly different according to Tukey’s HSD test (*p* < 0.05). (**b**) Graphical representation of results with pictures of petri dishes at the end of the diffusion assay.

**Figure 3:**
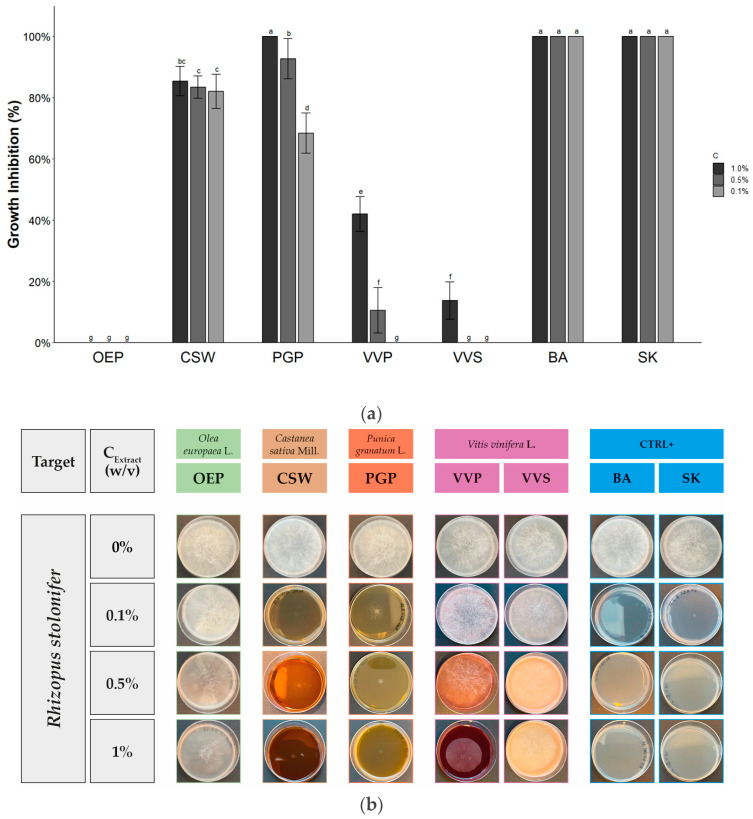
Antifungal activity of phenolic-rich extracts against *Rhizopus stolonifer*. (**a**) Histogram reporting the growth inhibitory activity of the extracts at different concentrations. Data expressed by mean ± SD (*n* = 6). Bars with different letters are significantly different according to Tukey’s HSD test (*p* < 0.05). (**b**) Graphical representation of results with pictures of petri dishes at the end of the diffusion assay.

**Figure 4:**
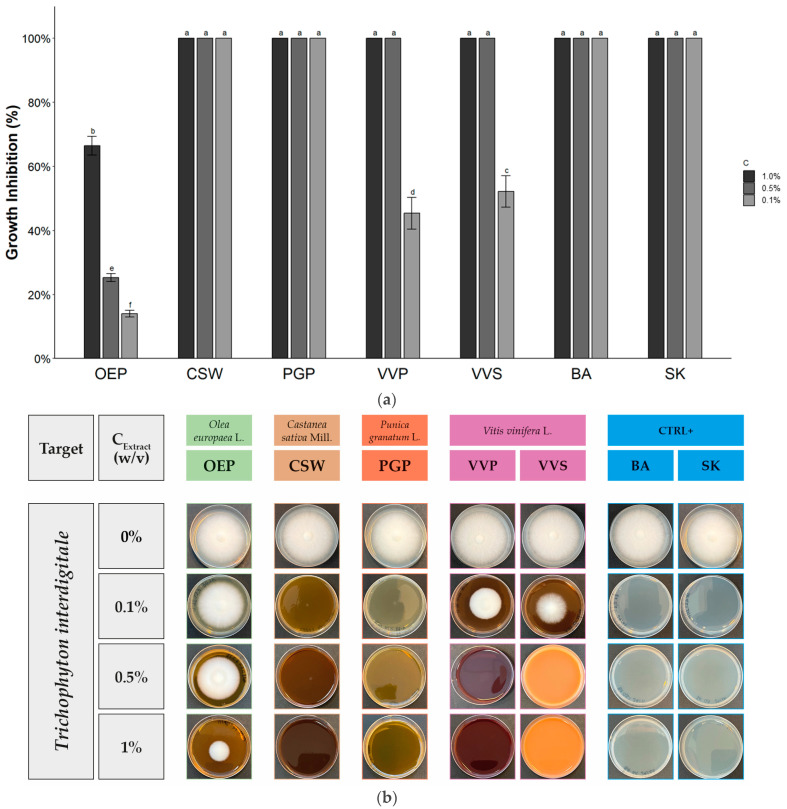
Antifungal activity of phenolic-rich extracts against *Trichophyton interdigitale.* (**a**) Histogram reporting the growth inhibitory activity of the extracts at different concentrations. Data expressed by mean ± SD (*n* = 6). Bars with different letters are significantly different according to Tukey’s HSD test (*p* < 0.05). (**b**) Graphical representation of results with pictures of petri dishes at the end of the diffusion assay.

The data of the antifungal activity of OEP, CSW, PGP, VVP, and VVS against *Alternaria* sp., *Aspergillus brasiliensis*, *Rhizopus stolonifer*, and *Trichophyton interdigitale* are reported in Table 3, Table 4 and Table 5 and Figure 1, Figure 2, Figure 3 and Figure 4.

As showed in Figure 1, OEP, CSW, PGP, and VVS did not reveal any inhibitory activity against *Aspergillus brasiliensis* also at 1.0% (*w*/*v*). The only extract exhibiting an activity, albeit modest, was VVP, with a growth inhibition of 48.0 ± 3.9% at 1.0% *w*/*v*, but a dramatic decrease in activity was observed at 0.5 and 0.1% *w*/*v*. Similar to other dark septate endophytes, some *Aspergillus* species, including A. *niger*, can degrade tannins using tannase enzymes. In particular, the *Aspergillus niger* GH1 strain showed the ability to degrade ellagitannins from pomegranate peel due to the activity of the ellagitannase enzyme, releasing ellagic acid from punicalagin [67,68,69]. The growth inhibition observed with VVP could be related to the presence of anthocyanins [70].

Table 3 and Figure 2 report the antifungal activity of OEP, CSW, PGP, VVP, and VVS against *Alternaria* sp. At 1.0% *w*/*v*, all extracts inhibited the growth of the pathogen, even if some differences of activity were observed. The lowest growth inhibition was evidenced for VVS (17.6 ± 8.0%) and the highest for CSW and PGP (100%). The inhibitory effect of CSW drastically decreased starting from 0.5% *w*/*v* while PGP retained the maximum activity also at 0.1% *w*/*v*. Due to this high effect, PGP was tested at a lower concentration (0.01% *w*/*v*), evidencing a significant decrease in activity, with an EC_50_ value of 0.026%, corresponding to 260 mg/L of extract and 29.9 mg/L of polyphenols. These data are in accordance with the literature [71,72]. In fact, sweet chestnut offers different waste matrices with antimicrobial activity against *Alternaria*. An aqueous extract from burs rich in hydrolysable tannins affects mycelial growth and spore germination against *Alternaria alternata* [71]. The main phenolic component of PGP, punicalagin, has been proved the most efficient pomegranate peel compound against *Alternaria alternata* AL19, with an inhibitory activity starting from 92.9 µM [72]. Satisfactory activity was observed for VVP at 1.0% *w*/*v* (62.6 ± 9.9%), which decreased proportionally with the concentration up to 34.7 ± 8.8% at 0.1% *w*/*v*. This activity was quantified to an EC_50_ value of 0.37% (3.7 g/L of the extract, 1.6 g/L of polyphenols), lower than CSW (0.54%, 5.4 g/L of the extract, 1.4 g/L of polyphenols). As expected, both BA and SK completely inhibited fungal growth from 1.0% to 0.1% *w*/*v*. The results of OEP at 0.1% *w*/*v* were influenced by guttation, which consists of the production of fungal exudates composed by liquid droplets. It caused a reduced growth of the mycelium, resulting in a fake enhanced antimicrobial activity and a high variability of data (18.2 ± 14.4%). However, during guttation, fungal species could produce secondary metabolites, including phenolic compounds and toxins involved in several key ecological roles [73].

As depicted in Table 4 and Figure 3, the antifungal activity of OEP, CSW, PGP, VVP, and VVS against *Rhizopus stolonifer* depended on the plant materials. At 1.0% *w*/*v*, OEP did not inhibit the growth; VVS and VVP produced a modest effect (13.7 ± 6.2 and 42.0 ± 5.7%, respectively); CSW showed significant activity (82.0 ± 8.7%); and PGP a total inhibitory effect. These last extracts also retained the activity at lower concentrations (0.5 and 0.1% *w*/*v*) with 83.4 ± 3.7% and 82.1 ± 5.5%; 92.8 ± 6.6% and 68.4 ± 6.6% of growth inhibition, respectively. The antifungal activity of pomegranate aqueous peel extract from the “shishe kab” Iranian cultivar against *Rhizopus stolonifer* was previously recorded using the poisoned food technique [74].

Table 5 and Figure 4 evidenced that all extracts exhibited a relevant growth inhibition against *Trichophyton interdigitale*. Except for OEP, which showed a growth inhibition of 66.5 ± 2.9% at 1.0% *w*/*v* [75,76], the extracts evidenced total inhibition. To the best of our knowledge, this is the first work investigating the antimicrobial activities of extracts of *Castanea sativa* Mill. against dermatophytes. No growth was observed using CSW at concentrations ranging from 1.0% to 0.1%, showing the best performance compared to both extracts and controls. With this extract, the test was carried out at lower concentrations. Only at 0.005% *w*/*v* was a drastic decay of antimicrobial activity recorded, resulting in an EC_50_ value of 0.0063%, corresponding to 63 mg/L of extract and 16.38 mg/L of polyphenols. PGP completely inhibited fungal growth until 0.1%, with an EC_50_ value of 0.014%, corresponding to 140 mg/L of extract 16.1 mg/L of polyphenols. In the literature, it was already reported that hydrolysable tannins possess antifungal activity against dermatophytes [77]. Crude extracts of pomegranate peels, as well as isolated punicalagin, showed inhibitory activity on conidia germination and mycelium growth of dermatophytes [78]. A strong performance was revealed even for VVS, in accordance with other works that highlighted the inhibitory effects of flavan-3-ols from different *Vitis vinifera* L. matrices against dermatophytes [79].

## 3. Materials and Methods

### 3.1. Chemicals

All solvents for HPLC-DAD-MS analyses (HPLC grade), formic acid, and epigallocatechin gallate (analytical grade) were purchased from Sigma Aldrich Chemical Company Inc. (Milwaukee, WI, USA). Tyrosol, gallic acid, ellagic acid, catechin, and malvidin 3-O-glucoside were supplied by Extrasynthèse S.A. (Lyon, Nord-Genay, France). Hydroxytyrosol was synthetized in the laboratory [80]. HPLC-grade water was obtained via distillation and purification with a Labconco Water Pro PS polishing station (Labconco Corporation, Kansas City, MO, USA). Potassium sorbate, benzoic acid, potassium hydroxide, and hydrochloric acid 37% were purchased from Carlo Erba Reagents Srl (Cornaredo, Milan, Italy), Tween 20 from Biolife Italiana Srl (Milan, Italy), and Potato Dextrose Agar from Oxoid Ltd. (Basingstoke, Hampshire, UK). Sterile plasticware and cotton swabs were purchased from Unifo Srl (Zero Branco, Treviso, Italy).

### 3.2. Phenolic-Rich Extracts

All extracts were derived from circular economy processes and were obtained by sustainable extraction methodologies. OEP was furnished by Bionap Srl (Piano Tavola Belpasso, Italy) and CSW was a gift of Gruppo Mauro Saviola Srl (Viadana, Italy). The commercially available Saviotan^®^ Feed was produced in the plant operating in Radicofani (Italy) by a sustainable, green process of hot water extraction and concentration/purification by membrane technology, described by us in a previous paper [37]. PGP was obtained from pomegranate peel (*Punica granatum* L., cv. Wonderful) collected in Grosseto, Italy. The peels were separated from the fresh fruits, finely chopped, put in a polypropylene filter bag, and then extracted for 1 h in boiling water (10% *w*/*v*) under magnetic stirring. The extraction mixture was left to cool down to room temperature and kept under maceration for 24 h; then, the extract was filtered under vacuum, frozen at −20 °C and lyophilized to obtain the final powder (yield: 9.2%). VVP was obtained from dried grape pomace furnished by Cantina Cesarini Sartori (Loc. Purgatorio, Gualdo Cattaneo, Italy). The powder (60 g) was extracted with ethanol/water = 70:30 (300 mL) adjusted at pH 2.5 by adding HCOOH for 24 h, under mechanical stirring. After filtration under vacuum, the solvent was evaporated; finally, the extract was rinsed with distilled water, frozen at −20 °C and lyophilized to obtain the final powder (yield: 0.9%). VVS was furnished by Consulente Enologica Srl (Pietraia di Cortona, Italy).

### 3.3. Characterization of Phenolic-Rich Extracts

OEP, CSW, PGP, VVP, and VVS were analyzed by HPLC-DAD-MS using a HP-1260 liquid chromatograph equipped with a DAD detector and an MSD API-electrospray (Agilent Technologies, Santa Clara, CA, USA) operating in negative and positive ionization mode. Mass spectrometer operating conditions were the following: gas temperature 350 °C at a flow rate of 10.0 L/min, nebulizer pressure 30 psi, quadrupole temperature 30 °C, and capillary voltage 3500 V. The fragmentor was set at 120 eV. For CSW and PGP, a Luna, C18 250 × 4.60 mm, 5 μm column (Phenomenex, Torrance, CA, USA) operating at 26 °C was used. The eluents were H_2_O (adjusted to pH 3.2 with HCOOH) and CH_3_CN. A four-step linear solvent gradient starting from 100% H_2_O up to 100% CH_3_CN was performed with a flow rate of 0.8 mL/min over a 55 min period, as previously described [37,45]. Gallic acid, flavanols, and procyanidins of VVP, VVS, and OEP were analyzed by using a column Lichrosorb RP18 250 × 4.60 mm i.d, 5 µm (Merck Darmstadt, Germany). The eluents were H_2_O adjusted to pH 3.2 with HCOOH and CH_3_CN. A four-step linear solvent gradient was used, starting from 100% H_2_O up to 100% CH_3_CN, for 117 min at a flow rate of 0.8 mL/min [25]. For anthocyanins of VVP, a Luna, C18 250 × 4.60 mm, 5 μm column (Phenomenex, Torrance, CA, USA) operating at 26 °C was used. The eluents were H_2_O (adjusted to pH 1.8 with HCOOH) and CH_3_CN. A multi-step linear solvent gradient was used, starting from 95% H_2_O up to 100% CH_3_CN, for 26 min at a flow rate of 0.8 mL/min.

Polyphenols present in the extracts were identified by using their chromatographic, spectrophotometric, and spectrometric data. Their retention times and data from HPLC-DAD and HPLC-MS were compared with those of the available specific commercial standards, also taking into account our previous results obtained by LC-MS-MS and/or LC-MS-TOF analysis of the same matrices, and data in the literature [81,82]. Each compound was quantified by HPLC-DAD using a five-point regression curve built with the available standards. Calibration curves with r^2^ ≥ 0.9998 were considered. The concentrations of the individual compounds were calculated by applying the appropriate corrections for changes in molecular weight. Ellagic acid and ellagitannins were calibrated at 254 nm with ellagic acid; gallic acid and gallotannins at 280 nm with gallic acid; epicatechin gallate (ECG) and epigallocatechin gallate (EGCG) at 280 nm with EGCG; catechin, epicatechin, and procyanidins at 280 nm with catechin hydrate; tyrosol and hydroxytyrosol at 280 nm with pure standards; and anthocyanins at 520 nm using malvidin-3-*O*-glucoside as reference. The evaluation of the polyphenol content was carried out in triplicate and the results were recorded as mean values with standard deviations ≤5%.

### 3.4. Fungal Pathogens

Extracts were tested against filamentous fungi *Aspergillus brasiliensis* (ex *A. niger*) derived from ATCC 16404, *Alternaria* sp. derived from ATCC 20,084 (Microbiologics, St. Cloud, MN, USA), *Rhizopus stolonifer* derived from ATCC 14,037, and *Trichophyton interdigitale* derived from ATCC 9533 (Kairosafe, Trieste, Italy). Fungi were maintained on Potato Dextrose Agar (PDA) at 25 °C.

### 3.5. In Vitro Antifungal Activity Assay

The fungal inoculum was prepared from fresh culture of about 4 days for *Rhizopus stolonifer*, 7 days for *Alternaria* sp. and *Aspergillus brasiliensis*, and 15 days for *Trichophyton interdigitale* following a procedure based on the EUCAST E.DEF 9.4 [83] with minor changes. Briefly, 5 mL of sterile water with Tween 20 (0.1% *v*/*v*) was added to the culture. To promote conidial suspension, the culture was gently scraped using a sterile cotton swab. The obtained suspension was recovered, shaken for about 15 s with a vortex and filtered to remove hyphae and clumps. The inoculum was spectrophotometrically adjusted to an equivalent final concentration of McFarland 0.5 (approximately 1–5 × 10^6^ CFU/mL).

Testing media were obtained by adding different amounts of extracts to PDA to obtain final concentrations of 1.0%; 0.5%; and 0.1% (*w*/*v*). As control, two food and cosmetic preservative SK and BA were used. After solubilization, pH was adjusted to 5.6 ± 0.2 using KOH 1 M or HCl 1 M. The media were then sterilized at 121 °C for 15 min and transferred to 55 mm petri dishes.

The antifungal activity assay was performed based on a diffusion method according to the literature with slight modifications [58,59]. A total of 10 μL of conidial suspension was inoculated in the center of the agar plate. Plates were incubated at 25 °C in darkness and growth was observed daily until the mycelium of the negative control (PDA only) touched the edge of the plate. Growth inhibition (GI), expressed as a percentage, was calculated by measuring the colony diameter and using the following equation:

GI(%) = [(*dc* − *dt*)/*dc*] × 100

where *dc* is the mean diameter of the negative control (PDA only) and *dt* is the mean diameter of the treatment.

### 3.6. Statistical Analysis

Data analysis was performed using RStudio Desktop (version 2023.13.0+386, Posit Software, PBC, Boston, MA, USA). To determine the differences between treatments, a one-way analysis of variance (ANOVA) was performed with significance level set at *p* = 0.05. Means separation was carried out using Tukey’s HSD test. EC_50_ was calculated using the “LL.2” function of the “drc” package, corresponding to a log-logistic model, where the lower limit was fixed at 0 (negative control *dc* for PDA only) and the upper limit was fixed at 1 (corresponding to total inhibition) [84].

## 4. Conclusions

In this paper, five phenolic-rich extracts (OEP, CSW, PGP, VVP, and VVS) derived from *Olea europaea* L. de-oiled pomace, *Castanea sativa* Mill. wood, *Punica granatum* L. peel, and *Vitis vinifera* L. pomace and seeds were characterized by HPLC-MS-DAD analysis to define their qualitative and quantitative phenolic profiles. Based on these analyses, OEP was found to be rich in hydroxytyrosol; CSW and PGP in hydrolysable tannins (vescalagin, castalagin, α-punicalagin, and β-punicalagin); and VVP and VVS in condensed tannins (procyanidins, trimers, and tetramers).

All extracts were tested against selected pathogenic filamentous fungi and dermatophytes such as *Aspergillus brasiliensis*, *Alternaria* sp., *Rhizopus stolonifer*, and *Trichophyton interdigitale* at three different concentrations (1.0%, 0.5%, and 0.1% *w*/*v*) using a diffusion assay. OEP, CSW, PGP, and VVS did not reveal any inhibitory activity against *Aspergillus brasiliensis* also at 1.0% (*w*/*v*). On the contrary, at the same concentration, VVP showed a (modest) growth inhibition. CSW and PGP exhibited a total growth inhibition against *Alternaria* sp. at 1.0% *w*/*v*, but VVP was also very effective at the same concentration. PGP retained the activity until 0.1% *w*/*v* with an EC_50_ value of 0.026%, corresponding to 260 mg/L of extract and 29.9 mg/L of polyphenols. Against *Rhizopus stolonifer,* PGP showed a total growth inhibition at 1.0% *w*/*v*, but strong performances were also observed at lower concentrations (up 0.1% *w*/*v*); CSW behaved similarly. Interestingly, CSW, PGP, VVS, and VVP showed a complete growth inhibition of *Trichophyton interdigitale* at 1.0% (*w*/*v*) and the activity was retained at lower concentrations. CSW was active until 0.005% *w*/*v* corresponding to an EC_50_ value of 0.0063% (63 mg/L of extract, 16.38 mg/L of polyphenols). PGP completely inhibited fungal growth until 0.1%, with an EC_50_ value of 0.014% (140 mg/L of extract, 16.1 mg/L of polyphenols).

Based on these data, we could conclude that the chemical composition is crucial for the biological activity of the extracts against the selected pathogens. In particular, hydrolysable and condensed tannins play a relevant role in the activity. The data obtained from this research seem promising for the potential application of some of these extracts as antifungal agents in the food and biomedical fields according to the green chemistry and circular economy concepts.

## Figures and Tables

**Table 1:** Quali-quantitative HPLC-DAD-MS analysis of OEP, CSW, and PGP.

Extract	Identification	RT (min)	λ_max_ (nm)	[M − H]^−^ (*m*/*z*)	mg/g
OEP	Hydroxytyrosol	20.6	280	153	138 ± 4.0
	Tyrosol	27.0	276	137	35.0 ± 0.8
	Total polyphenols				173 ± 5
CSW	Vescalin	6.9	246, 276 sh	631	9.3 ± 0.2
	Castalin	8.8	246, 280 sh	631	8.1 ± 0.2
	Pedunculagin I	11.5	258, 378 sh	783	10.0 ± 0.2
	Monogalloyl glucose	14.1	274	331	3.81 ± 0.08
	Gallic acid	15.4	272	169	16.2 ± 0.3
	Vescalagin	18.4	245, 280 sh	933	47.6 ± 0.5
	Dehydrated tergallic-C-glucoside	20.8	250, 374	613	9.3 ± 0.2
	Castalagin	21.9	248, 280 sh	933	97.7 ± 0.9
	Digalloyl glucose	24.1	274	483	19.6 ± 0.2
	Trigalloyl glucose	32.4	276	635	20.6 ± 0.2
	Tetragalloyl glucose	38.0	276	787	7.7 ± 0.1
	Ellagic acid	39.6	254, 370	301	6.1 ± 0.2
	Pentagalloyl glucose	40.8	274	939	4.26 ± 0.08
	Total polyphenols				260 ± 3
PGP	HHDP glucose 1	10.4	slope	481	0.75 ± 0.01
	HHDP glucose 2	11.1	slope	481	0.437 ± 0.009
	HHDP glucose 3	12.5	slope	481	0.71 ± 0.01
	Gallic acid	15.4	272	169	1.25 ± 0.02
	Monogalloyl glucose	15.5	274	331	0.106 ± 0.005
	α-Punicalin	17.0	258, 378	781	1.25 ± 0.04
	β-Punicalin	17.2	258, 380	781	1.32 ± 0.02
	Punicalagin isomer 1	18.4	258, 378	1083	6.90 ± 0.09
	Pedunculagin I	18.7	258, 378 sh	783	1.16 ± 0.06
	Punicalagin isomer 2	19.6	258, 378	1083	6.91 ± 0.08
	Pedunculagin III	21.0	260, 378	933	0.69 ± 0.01
	α-Punicalagin	23.7	258, 378	1083	27.1 ± 0.3
	β-Punicalagin	25.9	258, 380	1083	58.5 ± 0.6
	Ellagic acid hexoside	31.7	254, 362	463	2.10 ± 0.08
	Vanoleic acid bilactone	34.7	258, 366	469	0.45 ± 0.01
	Ellagitannin m/z 951	35.9	264, 364	951	1.02 ± 0.02
	Ellagic acid rhamnoside	37.0	254, 360	447	0.61 ± 0.03
	Ellagic acid pentoside	37.4	254, 362	433	0.87 ± 0.04
	Ellagic acid	39.1	254, 368	301	2.60 ± 0.08
	Total polyphenols				115 ± 2

Results are expressed as mg of each compound per g of extract. Retention times (RT), wavelengths of maximum UV absorbance (λ_max_), and the *m*/*z* values for the ESI-MS molecular ions after negative ionization of each compound are reported.

**Table 2:** Quali-quantitative HPLC-DAD-MS analysis of polyphenols in VVP and VVS.

Extract	Identification	RT (min)	λ_max_ (nm)	[M + H]^+^ (*m*/*z*)	mg/g
VVP	Delphinidin-3-glucoside	8.2	522	465	0.262 ± 0.007
	Cyanidin-3-glucoside	9.0	514	449	0.0097 ± 0.0003
	Petunidin-3-glucoside	9.3	524	479	0.365 ± 0.008
	Peonidin-3-glucoside	10.6	518	163	0.089 ± 0.003
	Malvidin-3-glucoside	11.0	526	493	1.30 ± 0.02
	Delphinidin-3-coumaroyl glucoside	15.8	530	611	0.130 ± 0.004
	Cyanidin-3-acetyl glucoside	17.6	524	491	0.0100 ± 0.0005
	Petunidin-3-coumaroyl glucoside	18.0	532	625	0.173 ± 0.005
	Malvidin-3-coumaroyl glucoside	20.0	532	639	0.80 ± 0.02
	Gallic acid	16.0	272	169 [M − H]^−^	2.37 ± 0.06
	Procyanidin dimer B3	30.6	280	579	7.0 ± 0.2
	Catechin	33.9	280	291	0.414 ± 0.008
	Procyanidin trimers	57.4	280	867	2.01 ± 0.05
	Procyanidin dimer B6	59.0	280	579	2.85 ± 0.08
	Procyanidin dimer B2	64.0	280	579	10.2 ± 0.3
	Epicatechin	76.5	280	291	0.320 ± 0.008
	Procyanidin trimer	77.0	280	867	50 ± 2
	Epicatechin gallate dimers	79.0	280	883	0.85 ± 0.02
	Procyanidin tetramers	90.9	280	1155	293 ± 4
	Epicatechin gallate dimers	104.4	280	883	53 ± 1
	Total polyphenols				425 ± 8
VVS	Gallic acid	16.0	272	169 [M − H]^−^	1.50 ± 0.02
	Procyanidin dimer B3	30.6	280	579	26 ± 1
	Catechin	33.9	280	291	45 ± 1
	Procyanidin trimer	57.4	280	867	8.8 ± 0.2
	Procyanidin dimer B6	59.0	280	579	11.2 ± 0.3
	Procyanidin dimer B2	64.0	280	579	13.6 ± 0.3
	Epicatechin	76.5	280	291	30.3 ± 0.8
	Procyanidin dimers gallate	88.3	280	731	20.1 ± 0.5
	Procyanidin trimers digallate	89.7	280	1171	315 ± 9
	Procyanidin tetramers (I)	90.0	280	1155	54.7 ± 0.16
	Epicatechin gallate	92.2	280	443	6.24 ± 0.08
	Procyanidin tetramers (II)	95.0	280	1155	11.6 ± 0.5
	Procyanidin dimers digallate	98.5	280	883	142 ± 5
	Total polyphenols				686 ± 20

Results are expressed as mg of each compound per g of extract. Retention times (RT), wavelengths of maximum UV absorbance (λ_max_), and the *m*/*z* values for the ESI-MS molecular ions after positive or negative ionization of each compound are reported.

**Table 3:** Antifungal activity of phenolic-rich extracts against *Alternaria* sp.

Extract/Compound	Growth Inhibition (%) at Different Extract Concentration (*w*/*v*)		
	1.0%	0.5%	0.1%
OEP	48.3 ± 2.7 ^c^	35.8 ± 8.7 ^bc^	18.2 ± 14.4 ^c^
CSW	100 ^a^	32.1 ± 7.9 ^c^	4.7 ± 4.4 ^c^
PGP	100 ^a^	100 ^a^	100 ^a^
VVP	62.6 ± 9.9 ^b^	46.3 ± 13.1 ^b^	34.7 ± 8.8 ^b^
VVS	17.6 ± 8.0 ^d^	24.1 ± 1.8 ^c^	5.0 ± 3.6 ^c^
BA	100 ^a^	100 ^a^	100 ^a^
SK	100 ^a^	100 ^a^	100 ^a^
Sign. code	***	***	***

Mean ± SD (*n* = 6). Values within each column followed by different letters are significantly different according to Tukey’s HSD test (*p* < 0.05). Sign. code expresses results of ANOVA analysis (*** corresponding to *p* < 0.001).

**Table 4:** Antifungal activity of phenolic-rich extracts against *Rhizopus stolonifer*.

Extract/Compound	Growth Inhibition (%) at Different Extract Concentration (*w*/*v*)		
	1.0%	0.5%	0.1%
OEP	No effect	No effect	No effect
CSW	82.0 ± 8.7 ^b^	83.4 ± 3.7 ^c^	82.1 ± 5.5 ^b^
PGP	100 ^a^	92.8 ± 6.6 ^b^	68.4 ± 6.6 ^c^
VVP	42.0 ± 5.7 ^c^	7.0 ± 7.9 ^de^	No effect
VVS	13.7 ± 6.2 ^d^	No effect	No effect
BA	100 ^a^	100 ^a^	100 ^a^
SK	100 ^a^	100 ^a^	100 ^a^
Sign. code	***	***	***

Mean ± SD (*n* = 6). Values within each column followed by different letters are significantly different according to Tukey’s HSD test (*p* < 0.05). Sign. code expresses results of ANOVA analysis (*** corresponding to *p* < 0.001).

**Table 5:** Antifungal activity of phenolic-rich extracts against *Trichophyton interdigitale*.

Extract/Compound	Growth Inhibition (%) at Different Extract Concentration (*w*/*v*)		
	1.0%	0.5%	0.1%
OEP	66.5 ± 2.9 ^b^	25.3 ± 1.2 ^b^	14.0 ± 1.0 ^d^
CSW	100 ^a^	100 ^a^	100 ^a^
PGP	100 ^a^	100 ^a^	100 ^a^
VVP	100 ^a^	100 ^a^	45.3 ± 4.9 ^c^
VVS	100 ^a^	100 ^a^	52.2 ± 4.9 ^b^
BA	100 ^a^	100 ^a^	100 ^a^
SK	100 ^a^	100 ^a^	100 ^a^
Sign. code	***	***	***

Mean ± SD (*n* = 6). Values within each column followed by different letters are significantly different according to Tukey’s HSD test (*p* < 0.05). Sign. code expresses results of ANOVA analysis (*** corresponding to *p* < 0.001).

## Data Availability

The data presented in this study are available on reasonable request from the corresponding authors. OPPURE The authors confirm that the data supporting the findings of this study are available within the article and Supplementary Materials.

## Supplementary Materials

The following supporting information can be downloaded at: https://www.mdpi.com/article/10.3390/molecules28114374/s1. Figure S1. Chromatographic profile of OEP at 280 nm; Table S1. Quali-quantitative analysis of OEP; Figure S2. Chromatographic profile of CSW at 254 and 280 nm; Table S2. Quali-quantitative analysis of CSW; Figure S3. Chromatographic profile of PGP at 254 and 280 nm; Table S3. Quali-quantitative analysis of PGP; Figure S4. Chromatographic profile of VVP at 520 and 280 nm; Table S4. Quali-quantitative analysis of VVP; Figure S5. Chromatographic profile of VVS acquired at 280 nm; Table S5. Quali-quantitative analysis of VVS.

## Sample Availability

OEP, CSW, PGP, VVP, and VVS are available from the authors.
