# Supplementary material for: Phenolic-Rich Extracts from Circular Economy: Chemical Profile and Activity against Filamentous Fungi and Dermatophytes
Source: Molecules. 2023 May 26;28(11):4374. doi: 10.3390/molecules28114374 (PMC10254516; doi:10.3390/molecules28114374)
Supplement: Supplementary file 1 [file molecules-28-04374-s001.zip › molecules-2399579-supplementary.pdf]

Supplementary Material

Phenolic-Rich Extracts from Circular Economy: Chemical Profile and Activity against Filamentous Fungi and Dermatophytes

Abstract. Fungal infections represent a relevant issue in agri-food and biomedical fields because they could compromise quality of food and humans' health. Natural extracts represent a safe alternative to synthetic fungicides and in the green chemistry and circular economy scenario, agro-industrial wastes and by-products offer an eco-friendly source of bioactive natural compounds. In this paper, phenolic-rich extracts from *Olea europaea* L. de-oiled pomace, *Castanea sativa* Mill. wood, *Punica granatum* L. peel, *Vitis vinifera* L. pomace and seeds were characterized by HPLC-MS-DAD analysis. Finally, these extracts were tested as antimicrobial agents against pathogenic filamentous fungi and dermatophytes as *Aspergillus brasiliensis*, *Alternaria* sp., *Rhizopus stolonifer* and *Trichophyton interdigitale*. The experimental results evidenced that all extracts exhibited a significant growth inhibition for *Trichophyton interdigitale*. *Punica granatum* L., *Castanea sativa* Mill., and *Vitis vinifera* L. extracts showed a high activity against *Alternaria* sp. and *Rhizopus stolonifer*. These data resulted promising for the potentiality of applications of some of these extracts as antifungal agents for food and biomedical fields.

Figures and Tables

Figure S1. Chromatographic profile of OEP at 280 nm.
Table S1. Quali-quantitative analysis of OEP.

Figure S2. Chromatographic profile of CSW at 254 and 280 nm.
Table S2. Quali-quantitative analysis of CSW.

Figure S3. Chromatographic profile of PGP at 254 and 280 nm.
Table S3. Quali-quantitative analysis of PGP.

Figure S4. Chromatographic profile of VVP at 520 and 280 nm.
Table S4. Quali-quantitative analysis of VVP.

Figure S5. Chromatographic profile of VVS acquired at 280 nm.
Table S5. Quali-quantitative analysis of VVS.

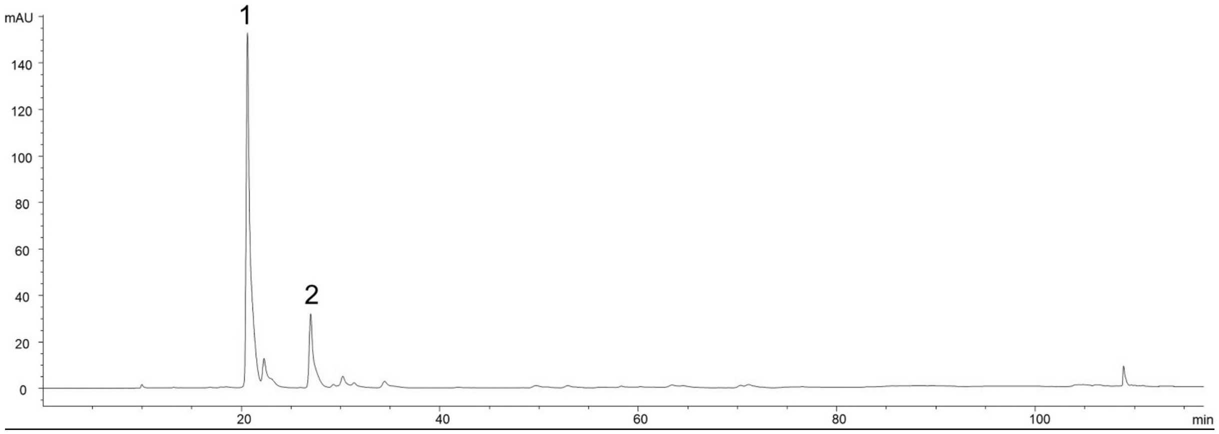

Figure S1. Chromatographic profile of OEP at 280 nm.

Table S1. Quali-quantitative analysis of OEP. The individual compounds are numbered as in **Figure S1**.

Identification	RT (min)	λ_{max} (nm)	$[M-H]^-$ (m/z)	mg/g
1. Hydroxytyrosol	20.6	280	153	138 ± 4.0
2. Tyrosol	27.0	276	137	35.0 ± 0.8
Total polyphenols				173 ± 5

Results are expressed as mg of each compound per g of extract. Retention times (RT), wavelengths of maximum UV absorbance (λ_{max}) and the m/z values for the ESI-MS molecular ions after negative ionization of each compound are reported.

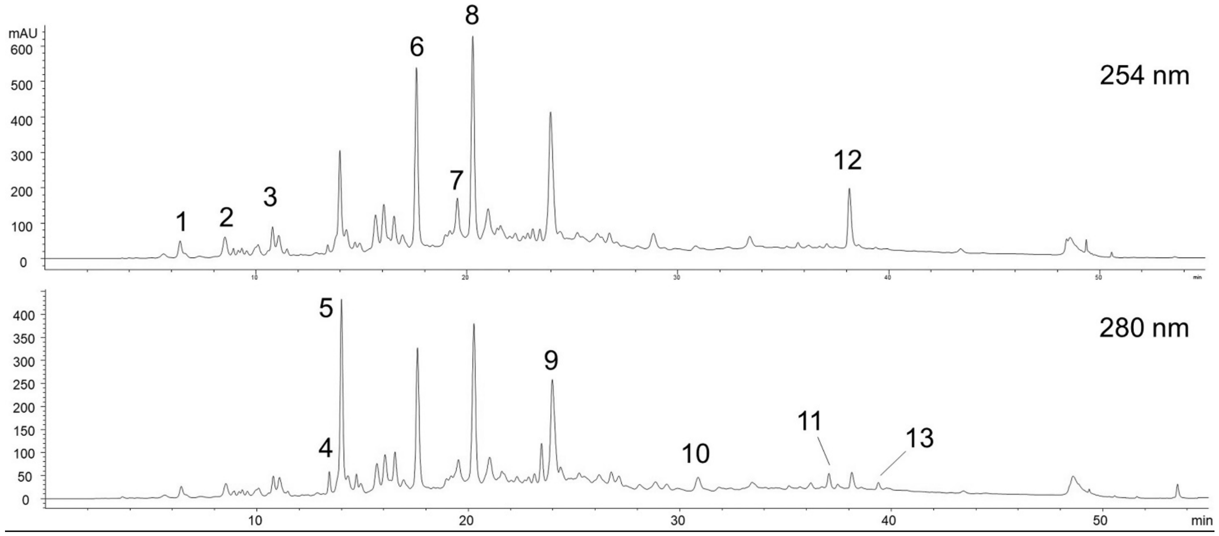

Figure S2. Chromatographic profile of CSW at 254 and 280 nm.

Table S2. Quali-quantitative analysis of CSW. The individual compounds are numbered as in **Figure S2**.

Identification	RT (min)	λ_{max} (nm)	$[M-H]^-$ (m/z)	mg/g
1. Vescalin	6.9	246, 276sh	631	9.3 ± 0.2
2. Castalin	8.8	246, 280sh	631	8.1 ± 0.2
3. Pedunculagin I	11.5	258, 378sh	783	10.0 ± 0.2
4. Monogalloyl glucose	14.1	274	331	3.81 ± 0.08
5. Gallic acid	15.4	272	169	16.2 ± 0.3
6. Vescalagin	18.4	245, 280 sh	933	47.6 ± 0.5
7. Dehydrated tergallic-C-glucoside	20.8	250, 374	613	9.3 ± 0.2
8. Castalagin	21.9	248, 280 sh	933	97.7 ± 0.9
9. Digalloyl glucose	24.1	274	483	19.6 ± 0.2
10. Trigalloyl glucose	32.4	276	635	20.6 ± 0.2
11. Tetragalloyl glucose	38.0	276	787	7.7 ± 0.1
12. Ellagic acid	39.6	254, 370	301	6.1 ± 0.2
13. Pentagalloyl glucose	40.8	274	939	4.26 ± 0.08
Total polyphenols				260 ± 3

Results are expressed as mg of each compound per g of extract. Retention times (RT), wavelengths of maximum UV absorbance (λ_{max}) and the m/z values for the ESI-MS molecular ions after negative ionization of each compound are reported.

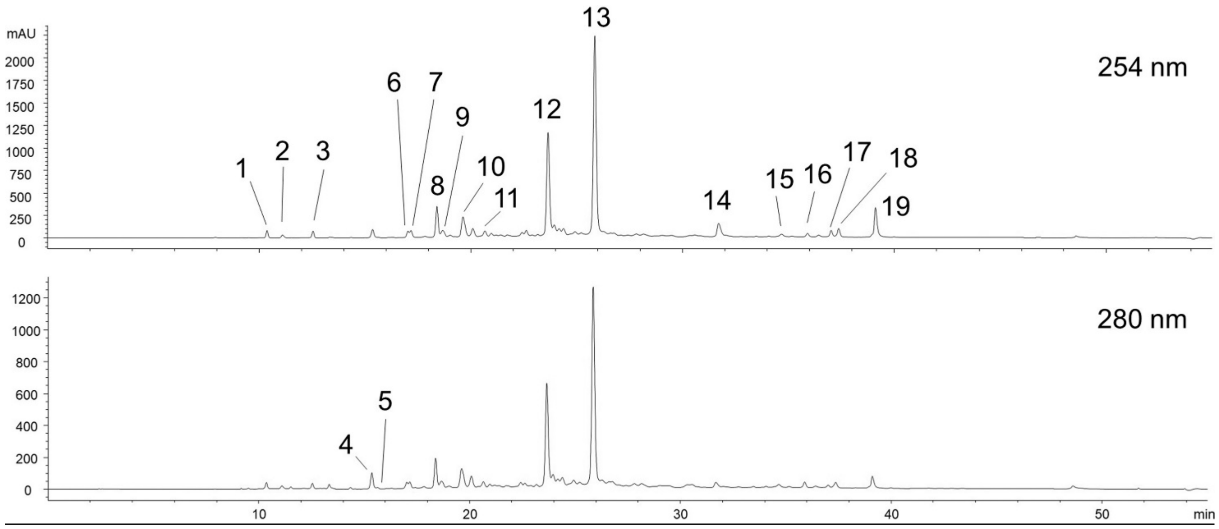

Figure S3. Chromatographic profile of PGP at 254 and 280 nm.

Table S3. Quali-quantitative analysis of PGP. The individual compounds are numbered as in **Figure S3**.

Identification	RT (min)	λ_{max} (nm)	$[M-H]^-$ (m/z)	mg/g
1. HHDP glucose 1	10.4	slope	481	0.75 ± 0.01
2. HHDP glucose 2	11.1	slope	481	0.437 ± 0.009
3. HHDP glucose 3	12.5	slope	481	0.71 ± 0.01
4. Gallic acid	15.4	272	169	1.25 ± 0.02
5. Monogalloyl glucose	15.5	274	331	0.106 ± 0.005
6. α-Punicalin	17.0	258, 378	781	1.25 ± 0.04
7. β-Punicalin	17.2	258, 380	781	1.32 ± 0.02
8. Punicalagin isomer 1	18.4	258, 378	1083	6.90 ± 0.09
9. Pedunculagin I	18.7	258, 378sh	783	1.16 ± 0.06
10. Punicalagin isomer 2	19.6	258, 378	1083	6.91 ± 0.08
11. Pedunculagin III	21.0	260, 378	933	0.69 ± 0.01
12. α-Punicalagin	23.7	258, 378	1083	27.1 ± 0.3
13. β-Punicalagin	25.9	258, 380	1083	58.5 ± 0.6
14. Ellagic acid hexoside	31.7	254, 362	463	2.10 ± 0.08
15. Vanoleic acid bilactone	34.7	258, 366	469	0.45 ± 0.01
16. Ellagitannin *m/z* 951	35.9	264, 364	951	1.02 ± 0.02
17. Ellagic acid rhamnoside	37.0	254, 360	447	0.61 ± 0.03
18. Ellagic acid pentoside	37.4	254, 362	433	0.87 ± 0.04
19. Ellagic acid	39.1	254, 368	301	2.60 ± 0.08
Total polyphenols				115 ± 2

Results are expressed as mg of each compound per g of extract. Retention times (RT), wavelengths of maximum UV absorbance (λ_{max}) and the m/z values for the ESI-MS molecular ions after negative ionization of each compound are reported.

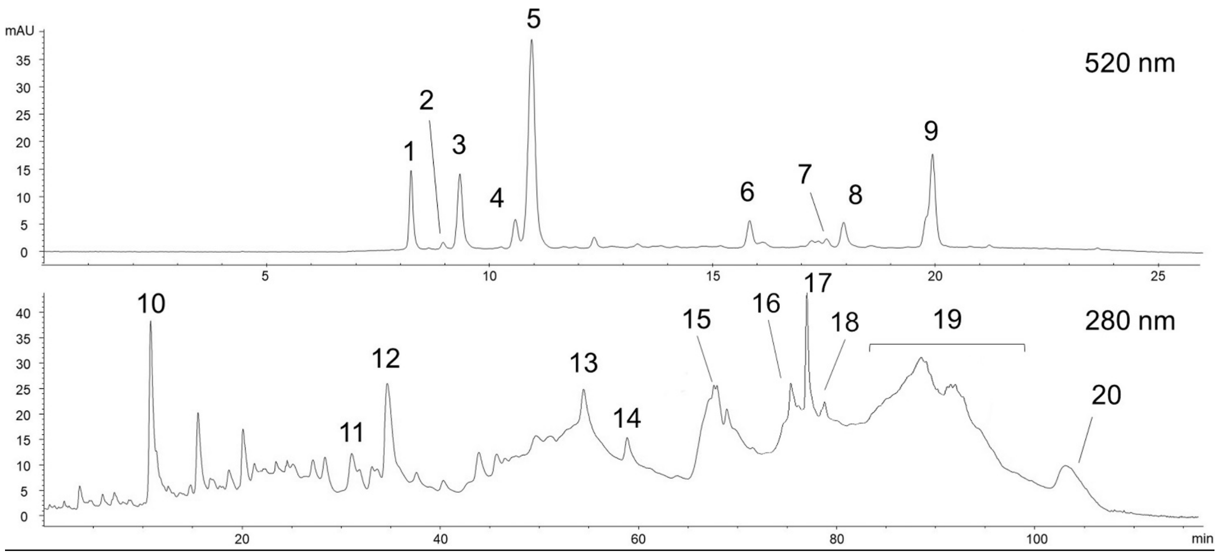

Figure S4. Chromatographic profile of VVP acquired at 520 and 280 nm.

Table S4. Quali-quantitative analysis of VVP. The individual compounds are numbered as in **Figure S4**.

Identification	RT (min)	λ_{max} (nm)	$[M+H]^+$ (m/z)	mg/g
1. Delphinidin-3-glucoside	8.2	522	465	0.262 ± 0.007
2. Cyanidin-3-glucoside	9.0	514	449	0.0097 ± 0.0003
3. Petunidin-3-glucoside	9.3	524	479	0.365 ± 0.008
4. Peonidin-3-glucoside	10.6	518	163	0.089 ± 0.003
5. Malvidin-3-glucoside	11.0	526	493	1.30 ± 0.02
6. Delphinidin-3-coumaroyl glucoside	15.8	530	611	0.130 ± 0.004
7. Cyanidin-3-acetyl glucoside	17.6	524	491	0.0100 ± 0.0005
8. Petunidin-3-coumaroyl glucoside	18.0	532	625	0.173 ± 0.005
9. Malvidin-3-coumaroyl glucoside	20.0	532	639	0.80 ± 0.02
10. Gallic acid	16.0	272	169 $[M-H]^-$	2.37 ± 0.06
11. Procyanidin dimer B3	30.6	280	579	7.0 ± 0.2
12. Catechin	33.9	280	291	0.414 ± 0.008
13. Procyanidin trimers	57.4	280	867	2.01 ± 0.05
14. Procyanidin dimer B6	59.0	280	579	2.85 ± 0.08
15. Procyanidin dimer B2	64.0	280	579	10.2 ± 0.3
16. Epicatechin	76.5	280	291	0.320 ± 0.008
17. Procyanidin trimer	77.0	280	867	50 ± 2
18. Epicatechin gallate dimers	79.0	280	883	0.85 ± 0.02
19. Procyanidin tetramers	90.9	280	1155	293 ± 4
20. Epicatechin gallate dimers	104.4	280	883	53 ± 1
Total polyphenols				425 ± 8

Results are expressed as mg of each compound per g of extract. Retention times (RT), wavelengths of maximum UV absorbance (λ_{max}) and the m/z values for the ESI-MS molecular ions after positive or negative ionization of each compound are reported.

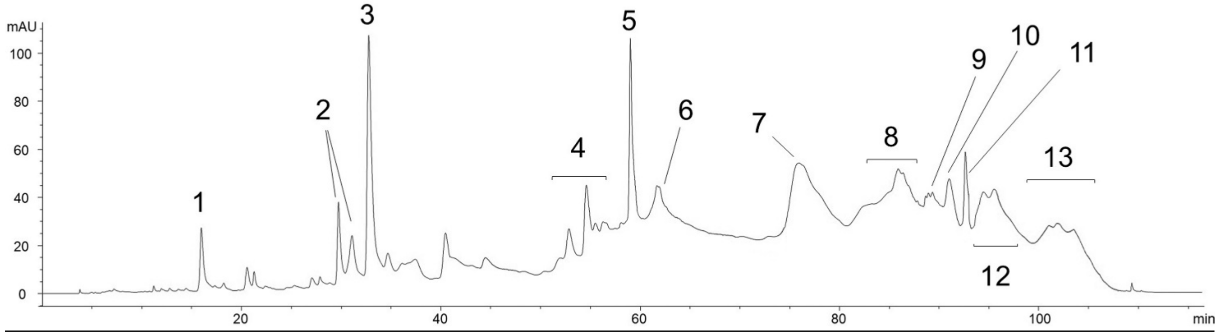

Figure S5. Chromatographic profile of VVS acquired at 280 nm.

Table S5. Quali-quantitative analysis of VVS. The individual compounds are numbered as in **Figure S5**.

**Identification**	**RT (min)**	**λ_{max} (nm)**	**$[M+H]^+$ (m/z)**	**mg/g**
1. Gallic acid	16.0	272	169 $[M-H]^-$	1.50 ± 0.02
2. Procyanidin dimer B3	30.6	280	579	26 ± 1
3. Catechin	33.9	280	291	45 ± 1
4. Procyanidin trimer	57.4	280	867	8.8 ± 0.2
5. Procyanidin dimer B6	59.0	280	579	11.2 ± 0.3
6. Procyanidin dimer B2	64.0	280	579	13.6 ± 0.3
7. Epicatechin	76.5	280	291	30.3 ± 0.8
8. Procyanidin dimers gallate	88.3	280	731	20.1 ± 0.5
9. Procyanidin trimers digallate	89.7	280	1171	315 ± 9
10. Procyanidin tetramers (I)	90.0	280	1155	54.7 ± 0.16
11. Epicatechin gallate	92.2	280	443	6.24 ± 0.08
12. Procyanidin tetramers (II)	95.0	280	1155	11.6 ± 0.5
13. Procyanidin dimers digallate	98.5	280	883	142 ± 5
Total polyphenols				686 ± 20

Results are expressed as mg of each compound per g of extract. Retention times (RT), wavelengths of maximum UV absorbance (λ_{max}) and the m/z values for the ESI-MS molecular ions after positive or negative ionization of each compound are reported.
